# Lysosomal Fusion Dysfunction as a Unifying Hypothesis for Alzheimer's Disease Pathology

**DOI:** 10.1155/2012/752894

**Published:** 2012-08-30

**Authors:** Kristen E. Funk, Jeff Kuret

**Affiliations:** Department of Molecular and Cellular Biochemistry, The Ohio State University College of Medicine, Columbus, OH 43210, USA

## Abstract

Alzheimer's disease is characterized pathologically by extracellular senile plaques, intracellular neurofibrillary tangles, and granulovacuolar degeneration. It has been debated whether these hallmark lesions are markers or mediators of disease progression, and numerous paradigms have been proposed to explain the appearance of each lesion individually. However, the unfaltering predictability of these lesions suggests a single pathological nidus central to disease onset and progression. One of the earliest pathologies observed in Alzheimer's disease is endocytic dysfunction. Here we review the recent literature of endocytic dysfunction with particular focus on disrupted lysosomal fusion and propose it as a unifying hypothesis for the three most-studied lesions of Alzheimer's disease.

## 1. Introduction

Alzheimer's disease (AD) is defined by the appearance of pathological hallmarks within specific areas of the brain, including amyloid plaques, composed of extracellular amyloid beta (A*β*) peptide [1] and neurofibrillary tangles (NFTs), composed of intracellular aggregates of the microtubule-associated protein tau [2]. Definitive diagnosis of AD is made at autopsy on the basis of these defining lesions, which have a well-documented but incompletely understood connection to disease. Although it has been debated whether these lesions are mediators of disease, they have clear correlation to disease progression and are useful as clues to understanding the pathological processes accompanying neurodegeneration. Additional hallmarks of AD include granulovacuolar degeneration (GVD), characterized morphologically as intracellular double membrane-bound organelles harboring a dense core and extracellular tau detectable in the cerebrospinal fluid (CSF) [3, 4]. Although the molecular mechanisms for individual pathological lesions have been proposed [5–7], a unifying hypothesis that rationalizes the appearance of all hallmarks of disease has been elusive. Here we review recent studies involving the intracellular vesicular pathways and their connection to the hallmark lesions of AD. We propose that vesicle trafficking dysfunction, and more specifically a failure in lysosomal fusion, may be the nidus of both defining lesions of AD as well as of GVD and extracellular tau, thus providing a unifying hypothesis for disease pathology.

## 2. Vesicle Trafficking Dysfunction

**Figure 1:**
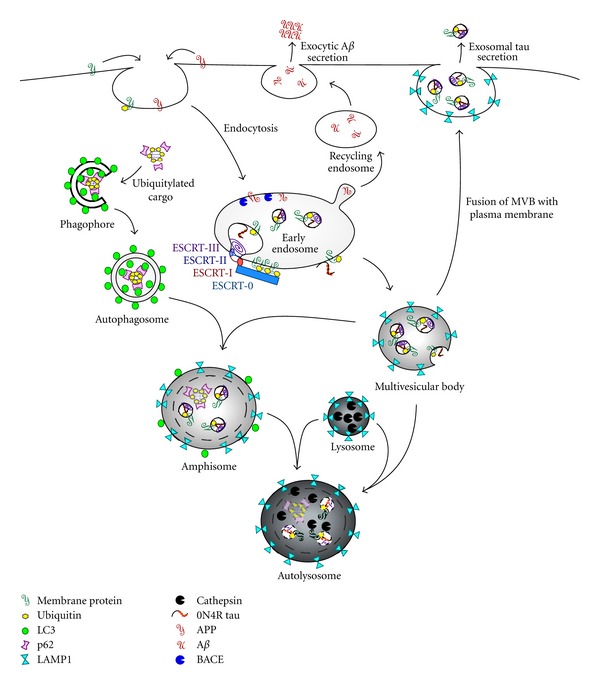
Intracellular vesicular trafficking pathways. Endocytosed surface proteins, such as APP, are delivered to the endosomal system by internalization. Internalized membrane proteins can be sorted into intralumenal vesicles of the MVB by sequential activity of ESCRTs 0, I, II, and III or delivered to the extracellular environment by the recycling endosome. Mature MVBs fuse with either the autophagosome of the autophagic pathway to form the amphisome or directly with the lysosome, which donates degradative hydrolases, creating the autolysosome where complete degradation of the sequestered material occurs. Alternatively, the multivesicular body can fuse with the plasma membrane, resulting in the exosomal secretion of the intralumenal vesicles and their internalized cargo.

Intracellular vesicle trafficking pathways form an interconnected, dynamic system for transfer of cellular constituents within the cell and between intracellular and extracellular compartments [8] (Figure 1). Cell surface proteins can enter the system through endocytosis. For example, the epidermal growth factor receptor is endocytosed and incorporated into early endosomes after binding its ligand and becoming ubiquitylated [9, 10]. Endocytosed material can then be budded off into recycling endosomes for exocytic release or incorporated into intralumenal vesicles of the multivesicular body (MVB) by the sequential activity of the endosomal sorting complexes required for transport (ESCRTs) [11–13]. Intralumenal vesicles and their contents may also be released as exosomes by fusion of the outer membrane of the MVB with the plasma membrane [14, 15]. The endocytic pathway merges with the macroautophagic (hereafter referred to as simply autophagic) pathway at either the early endosome or MVB stage [16]. In the autophagic pathway, target proteins are polyubiquitylated, fostering the binding of p62, the autophagic protein microtubule-associated protein 1 light chain 3 (LC3) [17], and the assembly of the autophagic membrane around the ubiquitylated cargo to form the autophagosome [18]. The autophagosome can then fuse with the lysosome directly or with the MVB to form the amphisome, a slightly acidic hybrid organelle. The amphisome also can fuse with the lysosome to form the autolysosome, a caustic organelle that degrades the enclosed proteins and organelles [19, 20]. 

 The dynamic nature of this system is difficult to capture in fixed *postmortem* tissue; however, there are several lines of evidence suggesting that changes in pathway flux are among the earliest pathologies observed in AD, preceding clinical symptoms of AD, intracellular NFT formation, and extracellular amyloid deposition [21, 22]. First, expression profiling during the progression of AD has revealed significant upregulation of effector genes of the early endosome (including *rab4* and *rab5*), the late endosome (*rab7*), and the exocytic pathway (*rab27*) [23–25]. These expression changes are consistent with morphological phenotypes observed in AD. For example, overexpression of rab5 causes enlarged endosomes, one of the earliest pathological alterations observed in AD [26]. Rab7 upregulation is found in vulnerable hippocampal basal forebrain regions but not in relatively spared striatum and cerebellum in mild cognitive impairment and AD [24, 27]. Second, the accumulation of A*β* and tau protein and the appearance of GVD bodies correlates with changes in trafficking pathways. These data are summarized below.

### 2.1. A*β* Accumulation

Full-length amyloid precursor protein (APP) is a transmembrane protein with an incompletely understood function. A*β*, the secreted peptide that aggregates to form senile plaques, is cleaved from APP by sequential activities of *β*- and *γ*-secretase enzymes [28]. *β*-secretase activity is catalyzed by multiple enzymes including BACE1, which contributes strongly to A*β* peptide production, BACE2, which is a BACE1 structural homolog, and lysosomal enzyme cathepsin B [29, 30]. Following *β*- cleavage, *γ*-secretase-mediated cleavage takes place within the transmembrane domain, yielding primarily 40 (A*β*_40_) or 42 (A*β*_42_) amino acid peptides [28]. A*β*_40_ is continuously and abundantly produced in both healthy and AD-affected brain, whereas A*β*_42_ is produced at lower levels in healthy individuals but is increased by familial AD (fAD-) causing mutations [31]. In general, CSF A*β*_42_, but not A*β*_40_, can serve as a surrogate biomarker for A*β* deposition in the brain [4, 32].

 Observations in both human tissue and cell culture implicate the endocytic pathway in A*β* production [22, 33–37]. AD-related endocytic dysfunction coincides with the detection of A*β* within endosomal compartments and autophagic vacuoles that collect within dystrophic neurites with the initial rise in soluble A*β* peptides [35, 36, 38]. The presence of A*β* in early endosomes also is consistent with the colocalization of APP and BACE1 within the same early endocytic compartments [39–42] and the degradation of BACE within the endosomal-lysosomal system [43]. One study suggests that internalized A*β* can aggregate within the cell and disrupt the vesicular membrane, thus contributing to its pathologic effect [44]. Intracellular trafficking of proteins involves a series of cytosolic factors, some of which are implicated in the regulation of APP trafficking and A*β* generation. For example, rab6, a protein implicated in membrane budding and clathrin, which mediates the internalization of APP from the cell surface, affect APP processing [45, 46]. Altogether, these data support a relationship between endocytic pathway dysfunction and the amyloidogenic processing of APP. 

 Rare disease-causing mutations have been discovered within the *APP *gene that result in increased A*β* production, A*β*42 : A*β*40 ratio, or A*β* aggregation rate [47]. More common, although still rare, are autosomal dominant mutations within the *PSEN1 *gene encoding presenilin 1(PS1), a transmembrane protein that acts as the catalytic subunit of the *γ*-secretase complex [48, 49]. This complex is located in the endoplasmic reticulum, transgolgi network, and endocytic compartments [50, 51]. The precise mechanism through which PS1 mutations drive AD is unclear, but they can cause aberrant processing of APP leading to increased A*β*42 : A*β*40 ratio [52, 53]. Further studies have revealed that familial AD-linked PS1 mutations significantly reduce budding from the endoplasmic reticulum and golgi, thereby decreasing delivery of APP to the cell surface [54]. This suggests that familial AD-linked PS1 variants increase A*β* production by decreasing intracellular transport of APP, thus prolonging the availability of APP for cleavage by *β*- and *γ*-secretases within the golgi. PS1 may regulate protein trafficking through its interaction with several cytosolic factors involved in the regulation of vesicular transport, such as rab11, rab6, and rab regulators [55–57]. Other data have ascribed the pathogenic effects of PS1 mutations to its role in facilitating maturation and targeting of a subunit of the vacuolar ATPase to the lysosome, which is essential for lysosomal acidification, protease activation, and degradation of lysosomal substrates [58]. This supports the impaired lysosomal fusion hypothesis, in that Bafilomycin A, a vacuolar ATPase inhibitor, is known to impair fusion of the lysosome with other membrane-bound vesicles [59]. However, recently the role of PS1 in the maturation of the vacuolar ATPase has been challenged, instead attributing lysosomal dysfunction in PS1-mutant cells to changes in gene expression associated with lysosome biogenesis [60] or alteration in lysosomal calcium storage and release [61]. Calcium storage and release have been proposed to regulate vesicular fusion [62], and in fact, is dependent on protonmotive force [63]. Therefore differentiating between these potential mechanisms may be challenging. In the future it will be important to determine the actual role of PS1 in the adult brain in order to parse these differences. Because A*β* is both generated and degraded via the endocytic and autophagic pathways [38], impaired lysosomal clearance could mediate PS1-dependent increases in A*β* concentration.

 The identification of genetic mutations that cause early-onset AD has lead to a greater understanding of the molecular mechanisms of disease; however, these mutations account for only a small fraction of cases. Rather, the majority of cases are sporadic with multiple susceptibility genes contributing incremental risk of developing disease [64, 65]. The role of each in AD pathogenesis will require further investigation; however, several of these susceptibility genes have known functions within or adjacent to the endocytic pathway. The strongest risk locus identified thus far is the gene that encodes apolipoprotein E (*APOE*), a protein component of lipoprotein particles that bind to cell surface receptors with function in lipid transport, A*β* trafficking, synaptic function, immune regulation, and intracellular signaling [66, 67]. A second risk-conferring gene is *SORL1*, a receptor that participates in trafficking vesicles from the cell surface to the Golgi-endoplasmic reticulum [68]. Other new risk genes, including *BIN1* [69] and *PICALM *[64], are involved in clathrin-mediated endocytosis. Clusterin, also known as apolipoprotein J, has also been identified to increase risk of AD [64, 65] and is hypothesized to act as an extracellular chaperone that influences A*β*-aggregation and receptor-mediated A*β* clearance by endocytosis [70, 71]. Although each of these genes confers only incremental risk, together they highlight the intracellular vesicle trafficking in the molecular pathogenesis of sporadic AD.

### 2.2. Tau

#### 2.2.1. Extracellular Tau

The appearance of tau and phospho-tau (p-tau) in CSF has been assumed to result from the passive release of tau from dying neurons [4]. However, recently it was shown that tau may be actively secreted into the extracellular compartment, where it is positioned to participate in the transmission of neurofibrillary pathology [72, 73]. Exosome-mediated release is considered a common, yet unconventional, mechanism responsible for the secretion of other aggregation-prone proteins including *α*-synuclein [74], prion protein [75], A*β* [76], and tau [77]. Secreted tau associates with both typical exosomal proteins, such as Alix and with proteins involved in tau misprocessing and AD pathogenesis, such as A*β*, presenilins, and fyn kinase [77]. Furthermore, exosomal tau is phosphorylated at Thr-181, accordant with CSF data [77]. Somewhat surprisingly, the greatest amount of exosomal tau was found in early AD, with the greatest being in Braak stage 3, progressively less so in later stages of AD when neuronal death is rampant and absent from non-AD controls diagnosed with nontauopathic dementias [77]. Because there is relatively little neurofibrillary degeneration at this early stage [78], the elevation of p-tau in AD CSF is not due to passive, nonspecific tau release consequent to neuronal death. Rather, these data highlight the vesicle trafficking pathway involved in tau processing and secretion. 

 A lysosomal fusion defect may account for the disease-related detection of tau in the CSF. The p-tau that accumulates where dendritic microtubules are being lost in dendrites is largely vesicle associated, and some of these vesicles are amphisome like, harboring both endocytic (fyn) and autophagic (LC3) markers [79]. Fyn activation during signal transduction typically causes the oligomerization and endocytosis of downstream elements [80] and targets at least some oligomerized targets of fyn phosphorylation to exosomes [81, 82]. Although in normal autophagy, LC3 is rarely exocytosed [83], the inability of the autophagic vesicle to fuse with the lysosome may alternatively shuttle vesicles towards the exosomal pathway (Figure 1). This exosomal secretion of tau corroborates the recent suggestion that interneuronal transfer of tau may be an important aspect of pathogenesis and account for the stereotypic neurofibrillary lesion progression [84–88]. Furthermore, this is consistent with recent evidence gathered from the lysosomal storage disorder, Niemann-Pick type C disease, in which exosomal release of cholesterol may serve as a cellular mechanism to partially bypass the traffic block that results in its toxic accumulation within the lysosome [89]. Altogether, although tau lacks the usual features of secreted proteins, such as an N-terminal hydrophobic “leader” sequence and lipidation sites, data suggests that tau is secreted, though it may be low in healthy neurons such as when the lysosomal system is functioning normally [73].

#### 2.2.2. Intracellular Tau

Failure of the lysosome to efficiently fuse with membrane-bound organelles and degrade encaptured proteins of the endocytic and autophagic pathways may also affect the intracellular concentrations of tau. Consistent with lysosome-mediated effects on tau levels, soluble tau is a substrate for lysosomal proteases such as cathepsin D *in vitro* and in cultured cells [90, 91], and inhibition of lysosome function with chloroquine can increase bulk tau levels [92]. Moreover, tau immunoreactivity associates with lysosomal protease cathepsin B in AD brain [93] and neuronal lysosomes in sections of both AD and control brain [94]. Because tau concentration modulates tau aggregation at both nucleation and extension steps [95, 96], it is a direct modulator of neurofibrillary lesion formation.

 Tau can be delivered to lysosomes through multiple pathways. First among these is autophagy, which can mediate turnover of tau phosphorylated on KXGS motifs in the microtubule repeat region [97, 98]. Evidence suggests that p-tau is less susceptible to proteasomal degradation mediated by hsp90 [99]. Therefore, under these conditions, failure of lysosome degradation could lead to elevated p-tau levels. Second, tau contains two sequence motifs for chaperone-mediated autophagy [99], a pathway that relies on hsc70 to target substrates directly to the lysosome. In a cellular model of tauopathy, the chaperone-mediated autophagy machinery and associated recognition motifs on tau protein enable the generation of tau fragments by lysosomal proteases [99]. These fragments have a higher propensity to form *β*-sheet aggregates that, once nucleated, can seed the aggregation of full length tau [100]. Because these fragments are generated via chaperone-mediated autophagy, they can be produced even if lysosomal fusion stalls so long as lysosomal proteases remain functional. Overall, the failure of the lysosomal pathway to efficiently clear intracellular tau may foster its pathological aggregation not only by increasing cytosolic concentrations but by generating aggregation-nucleating fragments as well. 

 Endocytic abnormalities may be involved in non-AD tauopathies, independent from A*β* deposition. Tau is redistributed to microtubule-poor regions of the cell when it is present in excess of available microtubule-binding capacity, which can result from overexpression, mutation, or posttranslational modifications (PTMs) that limit tau-microtubule binding [79, 101]. Since tau has been shown to modulate the activity of microtubule-associated motor proteins that mediate dendritic transport [102, 103], it is possible that toxicity resulting from tau accumulation at localized dendritic loci may have relevance to pathogenesis of non-AD tauopathies. Moreover, this toxic, vesicle-associated tau accumulates selectively in microtubule-poor segments containing organized microtubule bundles, suggesting that its accumulation is both the cause and consequence of localized microtubule destabilization [79]. Furthermore, neuropathological examination of Niemann-Pick-type C disease has revealed tau-laden NFTs without accompanying amyloid pathology [104]. Although the pathogenesis of the NFTs in Niemann-Pick-type C disease is not clear, the presence of dystrophic axonal swellings suggest cytoskeletal abnormalities [104]. This indicates that under certain conditions, lysosomal dysfunction alone can ultimately lead to NFT formation in the absence of A*β* deposits. 

#### 2.2.3. Mechanisms of Tau Regulation

The mechanisms regulating tau secretion through the intracellular vesicular trafficking pathways are unknown but may involve those also responsible for modulating pathological and normal tau biology, including alternative splicing and PTMs. Tau protein is encoded by a single gene comprising 16 exons [105, 106]. Exons 2, 3, and 10 undergo alternative splicing; however, exon 3 is expressed only in the presence of exon 2, thus yielding 6 isoforms. Depending on the presence or absence of the protein sequence encoded by exon 10 (e10), tau isoforms are called 4R (with e10) or 3R (without e10), referring to the number of imperfect microtubule-binding repeats. Similarly, tau isoforms are called 0N (without N-terminal inserts), 1N (with one N-terminal insert, encoded by exon 2), or 2N (with two N-terminal inserts, encoded by exons 2 and 3). E10 has received relatively more attention than either N-terminal inserts (e2 and e3) due to its role in microtubule binding and self-aggregation. However, recent evidence suggests that while extracellular secretion of tau requires the presence of an unknown element in the N-terminal domain [73], e2 specifically inhibits this secretion [72]. This agrees with known functions of the N-terminus of tau to mediate association with the plasma membrane and perimembranous structures [107]. 

 In conjunction with the inclusion or exclusion of e10, the affinity of tau for microtubules is regulated by its phosphorylation at sites in and around the microtubule-binding repeat region, with certain sites having more acute affects on the protein than others [108–110]. Exosomal fractions of conditioned media of human neuroblastoma cells are enriched in p-tau species associated with neurodegeneration [77]. Quantitative analysis has identified four epitopes within the proline-rich domain of tau that are most strongly enriched in secreted tau compared with intracellular tau [77]. These epitopes include AT270, AT8, AT100, and AT180, which correspond to phosphorylation at Thr-181, Ser-198 and Ser-202, Ser-210 and Thr-212, and Thr-231, respectively. Significantly, epitope AT270 (corresponding to phosphorylated Thr-181) is the epitope most highly enriched in secreted tau and is also an established biomarker for CSF-based diagnostics for early-stage AD [77, 111]. The potential role of phosphorylation as a regulator of tau secretion is corroborated by CSF biomarker data gathered from patients with acute brain injury, which at the cellular level involves axonal injury [112]. Importantly, total tau, but not p-tau, increases following acute brain injury [4], suggesting that while total tau may be released as a result of cellular damage and death, PTMs may actively regulate tau secretion. 

 As many as 30 different phosphorylation sites have been identified on tau [113], and although phosphorylation is the most comprehensively studied of the tau PTMs, filamentous tau is known to be extensively modified by several PTMs, including lysine-directed ubiquitylation [114]. We and others have identified at least three sites of ubiquitylation on tau [115–117]. Although most well known for its role in proteasomal degradation of proteins, ubiquitin is also the best known signal for endocytic sorting [118]. As a general rule, targeting to the proteasome requires attachment of a chain of at least four ubiquitins [119], whereas targeting to the endocytic pathway requires only a single ubiquitin or a short chain of two or three ubiquitins [118]. These ubiquitins are recognized by the ESCRT machinery, which sort the ubiquitylated cargo and direct it towards its destined pathway [12, 13]. In addition to the influence that PTMs exert on tau function individually, it is thought that various modifications may cooperate and compete in a coordinated fashion. For example, recently two modifications have been identified that are also lysine directed: acetylation [120, 121] and methylation [117]. Because these modifications both directly compete with ubiquitin for lysine site occupancy, there is potential for acetylation and methylation to directly affect the rate of turnover of tau protein and its motility through the endocytic pathway. Furthermore, in other biochemical pathways, such as histone-regulated processes, there is precedent for PTMs, including acetylation and methylation, to indirectly affect neighboring site modification, such as phosphorylation [122], though the potential for this type of modification crosstalk to be occurring in tau protein and its effect on tau metabolism has yet to be examined.

### 2.3. GVD Bodies

 Evidence of dysfunctional endocytic/autophagic pathway in AD extends to GVD. GVD body load increases with disease severity and episodic memory decline [3, 123, 124]. GVD body ultrastructure has been extensively studied, revealing an electron-dense core with coarse or vesicular morphology surrounded by a double-layered membrane [125]. Because of the two-layered membrane, it has long been hypothesized that these lesions are of autophagic origin [125]. Recent work by us and others has implicated the endocytic pathway in the formation of GVD bodies due to strong immunoreactivity of CHMP2B, a component of ESCRT-III, in the GVD body core [126, 127]. This work also suggests a failure of the MVB to fuse with the lysosome [126], which could account for an accumulation of autophagic intermediates [128] and an increased size and density of the MVB [10]. We hypothesize that consequently, flux is routed to the autophagic pathway, which results in the accumulation of abnormally large amphisome-like intermediates owing to the same lysosomal fusion defect combined with mTOR-mediated suppression of phagophore formation [129–131]. The genetic increase in *rab7* in AD [23, 25] is somewhat inconsistent with this hypothetical lysosomal fusion failure, though it is possible that this upregulation is acting in an ineffective compensatory manner. However, it is interesting that late endosome marker *rab24*, but not secretory protein *rab27*, is upregulated in CA1 pyramidal neurons [23, 25] considering that GVD most greatly affects the CA1 region of the hippocampus [3]. This may reflect alternative responses to the disease process with one cell type opting for increased secretion and another for increased degradation to remove the toxic protein.

## 3. Conclusions

 We propose that lysosome fusion dysfunction is a candidate nidus for the major pathological hallmarks of AD including both defining lesions of AD, GVD bodies, as well as the presence of tau in the CSF. Although the origin of fusion dysfunction is not yet fully understood, it most likely results from the convergence of multiple factors, like the disease it manifests. The involvement of lysosomal dysfunction as an underlying and unifying hypothesis for AD pathology may change the overall view of AD pathogenesis. It provides novel insights into disease-associated mechanisms of protein misprocessing and potentially new modes of disease progression. There is also potential clinical importance of tau secretion biomarkers for CSF-based diagnostics and for the direction of future disease-modifying therapeutics.

## Abbreviations

A*β*: Amyloid beta
AD: Alzheimer's disease
APP: Amyloid precursor protein
BACE: Beta secretase
CHMP2B: Charged multivesicular body protein 2B
CSF: Cerebrospinal fluid
e2, e3, e10: Protein sequence encoded by exons 2, 3, 10, respectively
ESCRT: Endosomal sorting complex required for transport
GVD: Granulovacuolar degeneration
LC3: Microtubule-associated protein 1 light chain 3
MVB: Multivesicular body
NFT: Neurofibrillary tangle
PS1: Presenilin 1
p-tau: Phosphorylated tau
PTM: Posttranslational modification.
